# Population pharmacokinetics of artesunate and amodiaquine in African children

**DOI:** 10.1186/1475-2875-8-200

**Published:** 2009-08-20

**Authors:** Kasia Stepniewska, Walter Taylor, Sodiomon B Sirima, Esperance B Ouedraogo, Alphonse Ouedraogo, Adama Gansané, Julie A Simpson, Caroline C Morgan, Nicholas J White, Jean-René Kiechel

**Affiliations:** 1 Mahidol-Oxford Tropical Medicine Research Unit, Faculty of Tropical Medicine, Mahidol University, 420/6 Rajvithi Road, Bangkok 10400, Thailand; 2 Centre for Clinical Vaccinology and Tropical Medicine, Churchill Hospital, Oxford, UK; 3 Service de Médecine Internationale et Humanitaire, Hopitaux Universitaires de Genève, Switzerland; 4 Centre National de Recherche et de Formation sur le Paludisme, Ouagadougou, Burkina Faso; 5 Groupe de Recherche Action en Santé (GRAS), Ouagadougou, Burkina Faso; 6 Centre for Molecular, Environmental, Genetic and Analytic Epidemiology, The University of Melbourne, Melbourne, Australia; 7 Cardinal Systems, Paris, France; 8 Drugs for Neglected Diseases Initiative, Geneva, Switzerland

## Abstract

**Background:**

Pharmacokinetic (PK) data on amodiaquine (AQ) and artesunate (AS) are limited in children, an important risk group for malaria. The aim of this study was to evaluate the PK properties of a newly developed and registered fixed dose combination (FDC) of artesunate and amodiaquine.

**Methods:**

A prospective population pharmacokinetic study of AS and AQ was conducted in children aged six months to five years. Participants were randomized to receive the new artesunate and amodiaquine FDC or the same drugs given in separate tablets. Children were divided into two groups of 70 (35 in each treatment arm) to evaluate the pharmacokinetic properties of AS and AQ, respectively. Population pharmacokinetic models for dihydroartemisinin (DHA) and desethylamodiaquine (DeAq), the principal pharmacologically active metabolites of AS and AQ, respectively, and total artemisinin anti-malarial activity, defined as the sum of the molar equivalent plasma concentrations of DHA and artesunate, were constructed using the non-linear mixed effects approach. Relative bioavailability between products was compared by estimating the ratios (and 95% CI) between the areas under the plasma concentration-time curves (AUC).

**Results:**

The two regimens had similar PK properties in young children with acute malaria. The ratio of loose formulation to fixed co-formulation AUCs, was estimated as 1.043 (95% CI: 0.956 to 1.138) for DeAq. For DHA and total anti-malarial activity AUCs were estimated to be the same. Artesunate was rapidly absorbed, hydrolysed to DHA, and eliminated. Plasma concentrations were significantly higher following the first dose, when patients were acutely ill, than after subsequent doses when patients were usually afebrile and clinically improved. Amodiaquine was converted rapidly to DeAq, which was then eliminated with an estimated median (range) elimination half-life of 9 (7 to 12) days. Efficacy was similar in the two treatments groups, with cure rates of 0.946 (95% CI: 0.840–0.982) in the AS+AQ group and 0.892 (95% CI: 0.787 – 0.947) in the AS/AQ group. Four out of five patients with PCR confirmed recrudescences received AQ doses < 10 mg/kg. Both regimens were well tolerated. No child developed severe, post treatment neutropaenia (<1,000/μL). There was no evidence of AQ dose related hepatotoxicity, but one patient developed an asymptomatic rise in liver enzymes that was resolving by Day-28.

**Conclusion:**

The bioavailability of the co-formulated AS-AQ FDC was similar to that of the separate tablets for desethylamodiaquine, DHA and the total anti-malarial activity. These data support the use this new AS-AQ FDC in children with acute uncomplicated falciparum malaria.

## Background

Anti-malarial drug resistance in *Plasmodium falciparum *in tropical countries has resulted in an increase in childhood morbidity and mortality, especially in African under fives, the most vulnerable group affected by falciparum malaria [1]. Many countries are now switching or have switched from ineffective monotherapies to reliably effective artemisinin-based combination therapy (ACT) following global recommendations by the World Health Organization (WHO) [2].

The rationale for using ACT is based on the concept that the artemisinin component will rapidly reduce parasitaemia leaving the residual parasitaemia to be cleared by high concentrations of the partner drug. In this way, treatment responses are rapid and reliable, and the probability of the development of *de novo *resistance is greatly reduced [2,3].

Rapid and effective treatment with ACT is considered essential by the WHO for rolling back malaria, but treatments are effective only if the full doses are taken. There is increasing acceptance that adherence is improved by the use of simplified, fixed dose combinations presented in easy to use packaging [3,4].

Artesunate-amodiaquine is one of four ACT currently recommended by the WHO and is adopted as the first line treatment in 18 African countries with a further three countries where it is one of the first-line treatments [5]. Until recently AS + AQ has been available only as a loose combination of the individual drugs, frequently in a co-blister pack. Since ACT should be formulated as fixed dose combinations (FDC), where possible [2], a co-formulated AS/AQ product has been developed by a partnership, lead by the Drugs for Neglected Diseases *initiative *(DND*i*) with the aim of making available an inexpensive, blister packaged, new, simplified FDC dosing regimen based on age. This new FDC has now been registered.

Although amodiaquine has been used extensively in Africa for many years, there are very limited paeadiatric and adult PK data. The present study was conducted as part of the clinical development and registration of this new FDC under well-established use. One key element was to show whether the FDC had similar bioavailablity to the presently used, loose AS + AQ regimen. Therefore, the FDC was tested against loose AS + AQ in children under five, with acute uncomplicated malaria in an open-label, randomized comparative trial conducted in Burkina Faso. The goal of the pharmacokinetic evaluation was to assess the comparative bioavailability of the new FDC and to characterize its pharmacokinetic properties.

## Methods

This study was a part of the efficacy and safety evaluation of new artesunate and amodiaquine (AS/AQ) FDC as compared to the same drugs given separately (AS+AQ). Results of the efficacy part of this evaluation have already been reported [6]. The trial was conducted in Burkina Faso, at the Healthcare District of Pissy between September and November 2006.

The study was approved by the Burkina Faso Ethics Committee for Health Research and the WHO Secretariat Committee on Research Involving Human Subjects and was carried out in accordance with the ICH Guidelines for Good Clinical Practice. Written informed consent was obtained from parents or guardians before patients began treatment. The trial is registered with the open clinical trial registry [7] under the identifier number ISRCTN07576538.

Patients of both sexes, between six months and five years of age, weighing = 5 kg with *Plasmodium falciparum *monoinfection of more than 1,000 parasites/μL and a measured fever (axillary temperature ≥ 37.5°C) were included in the study. Patients were excluded if they: (i) had features of severe and complicated malaria [8]; (ii) had taken the study drugs or other anti-malarial drugs within seven days before inclusion (or within three days if an artemisinin was used); or (iii) if they were receiving treatment with antibiotics with anti-malarial activity. A randomization list was computer generated. Individual treatment allocations were kept in sealed envelopes and opened after patients were admitted into the study. All treatments were administered by a study nurse. Fixed dose combination (AS/AQ) was administered according to a newly designed, age based dosing regimen [9] and loose dose combination (AS+AQ) according to the manufacturer's instructions for the Arsucam^® ^blister pack (Sanofi-Aventis, Paris, France). For both regimens, the target doses for artesunate and amodiaquine base were 4 mg/kg/day and 10 mg/kg/day, respectively, with, therapeutic ranges of 2 to 10 mg/kg/day and 7.5 to 15 mg/kg/day [9].

The FDC contained 25 mg of AS and 67.5 mg of AQ. The dose was one (age <12 months) or two (age of 12 to 60 months) tablets. The loose blister pack contained 50 mg tablets of AS (Arsumax^®^, Sanofi-Aventis) and 153 mg tablets of AQ (Flavoquine^®^, Sanofi-Aventis). The dose was half or one tablet of both drugs according to age, as described above. Patients remained in the health centre for an hour following each treatment administration. Vomiting during this one hour observation period resulted in re-administration of the same dose of the study drugs.

Following the first visit, patients were seen after 24, 48, and 72 hours, 7, 14, 21 and 28 days for clinical (symptoms, temperature, adverse effects) and parasitological assessments. Parasite density was determined by counting the number of asexual parasites per 200 leucocytes on a Giemsa-stained thick film and expressed as the number/μL, assuming a leukocyte count of 8,000/μL. If up to 500 parasites were counted before reaching 200 leucocytes, the counting process was stopped at the end of the last field. Gametocytes were counted and expressed as the number per 1,000 leucocytes (thick film). Routine haematology (Pentra 60^®^) and biochemistry (Hospitex Screen Master Tecno^®^) blood samples were taken and analysed on Days 0, 7 and 28.

Patients failing or not tolerating their treatment were withdrawn from the study and rescued with 25 mg/kg/day of oral or parental quinine base in three divided doses for 7 days. There was a systematic investigation of all patients lost to follow-up.

### Efficacy endpoints

The primary efficacy endpoint was the PCR corrected parasitological cure, assessed by Kaplan Meier survival analysis. The criteria for treatment failure followed broadly those of the WHO [8]: (i) signs of severe malaria or danger signs at any time during follow up, (ii) parasitaemia at Day-2 greater than parasitaemia at Day-0, (iii) Day-3 parasitaemia greater than or equal to 25% of parasitaemia measured on Day-0, (iv) parasitaemia on Day-7, and (v) a recurrent parasitaemia after Day-7 that was a PCR proven, recrudescent parasitaemia.

Patients with recurrent parasitaemia after Day-7 were classified as failure or new infections by analysing sequentially by PCR polymorphic segments of three parasite genes: first the merozoite surface protein (MSP) 2, then MSP 1 then glutamate rich protein (GLURP), according to a previously published method [10]. A new infection was diagnosed if the allelic pattern for any one of the loci differed completely between the baseline and recurrent samples. All other allelic patterns were diagnosed as a resistant infection.

Safety of treatment was assessed by collecting clinical (symptoms, signs) and routine laboratory data during follow up. The standard International Conference on Harmonisation (ICH) definitions of an adverse event and a serious adverse event were used [11]. Adverse events criteria were determined using the Common Toxicity Criteria (US NIH, CTC v2 1999) and graded as mild (1), moderate (2), severe (3) or very severe (4).

### Sample size and sampling

A sample size of 140 children was deemed adequate for the population PK model given the limitation of taking blood from small children. Children were divided into two groups of 70 (35 in each treatment arm). The first 70 recruited children formed the AS pharmacokinetics group. All children were sampled three times: (i) before the first dose, (ii) after the first dose, randomly at either 0.5, 1, 2 or 4 hours, (iii) after the third dose, randomly at either 0.5, 1, 2 or 4 hours. The second group (patients 71 to 140) participated in the study of AQ pharmacokinetics. All children were sampled four times: (i) before the first dose, (ii) at 4 hours after the 3^rd ^dose, (iii) on Day-7 or 14 of the follow-up period, (iv) on Day-21 or 28 of the follow-up period.

Each time, a sample of 1 mL of venous blood was collected in lithium heparin tubes. Plasma was separated immediately by centrifugation and stored frozen at approximately -20°C until sample analysis within three months.

### Analytical methods

The concentrations of AS and AQ and their respective pharmacologically active metabolites dihydroartemisinin and desethylamodiaquine in plasma samples collected during the study, were determined using sensitive and specific Liquid Chromatography/Mass Spectrometry/Mass Spectrometry (LC-MS/MS) methods developed and validated by PAREXEL International.

Artesunate, dihydroartemisinin and the internal standard (artesunate-D4) were separated from human plasma by solid-phase extraction (SPE) using Oasis HLB C18 extraction cartridges and analysed by reversed-phase LC-MS/MS in the Turbo Ion Spray positive mode. The assay was carried out using a 200 μL sampling volume of human plasma and the lower limit of quantification (LLOQ) of the LC-MS/MS method was 1.0 ng/mL for both artesunate and dihydroartemisinin. The coefficients of variations (CV%) during the analysis of artesunate and dihydroartemisinin were 11.3% and 6.5% at low (3 ng/mL), 3.4% and 5.0% at medium (100 ng/mL) and 3.9% and 9.0% at high (200 ng/mL) concentrations.

Amodiaquine, desethylamodiaquine and the internal standard (Amodiaquine-D10) were separated from human plasma by a different solid-phase extraction (SPE) using Oasis HLB C18 extraction cartridges and analysed by reversed-phase LC-MS/MS in the Turbo Ion Spray positive mode. The assay was carried out using a 200 μL sampling volume of human plasma and the lower limit of quantification (LLOQ) of the LC-MS/MS method was 1.0 ng/mL of amodiaquine and desethylamodiaquine. The CV% during the analysis of amodiaquine and desetylamodiaquine were 5.9% and 8.7% at low (3 ng/mL) and 6.3% and 8.0% at moderate (100 ng/mL) and 6.1% and 5.3% at high (200 ng/mL) concentrations.

Sample collection during this clinical study was performed in lithium heparin tubes and did not include stabilization with phenylmethyl sulfonylfluoride (PMSF). Long term stability of amodiaquine and desethylamodiaquine in human plasma samples collected on lithium heparin without PMSF was demonstrated for a 6-month period at approximately -20°C [12].

Long term stability of artesunate and dihydroartemisinin under these conditions [13] was demonstrated after 115 days of storage at -20°C. However, human plasma samples collected during this study were stored at -20°C for up to 147 days prior to last sample analysis. This period of storage is beyond the validated 16 weeks. Consequently, the long-term stability of AS and its pharmacologically active metabolite dihydroartemisinin in human plasma with lithium heparin as anti-coagulant and without PMSF was retested and demonstrated satisfactory stability for artesunate containing samples for 173 days and dihydroartemisinin for 142 days for samples prepared as described in the study.

The effects of haemolysis on the determinations were investigated during the respective validation studies and were found to have no effect on the assay of amodiaquine and desethylamodiaquine. However, the assay of AS and dihydroartemisinin was significantly affected in an unpredictable way by haemolysis.

Therefore, the presence of evident haemolysis observed in many of the plasma samples led to their classification as *not reportable *(no quantitative result) for AS and dihydroartemisinin.

### Pharmacokinetic analysis

Amodiaquine concentrations were usually low or not detectable. The population pharmacokinetics of desethylamodiaquine were modelled using the non-linear mixed effects approach. One and two compartment models were investigated. Due to the small number of detectable samples per subject (at most 3 per subject) and the timing of samples, the absorption rate constant was fixed (as 0.13 h^-1 ^from previously published work [14]) and at most two random effects which were not correlated could be fitted at the same time. Children with detectable AQ concentrations before the first dose were excluded from the analysis. Children with detectable desethylamodiaquine levels and corresponding negative AQ levels before the first dose were included in the population modelling, and a first order elimination process was assumed for these samples.

Pharmacokinetic parameters of DHA and the total anti-malarial activity, defined as the sum of the plasma levels of DHA and artesunate calculated in nmol/L units (taking molecular weight as 384.4 for artesunate and 284.9 for DHA) were also modelled (expressed as DHA equivalents) using the population approach as described previously [15]. This assumes equal anti-malarial activity. A one compartment model with three parameters (absorption rate constant, total apparent volume of distribution (V/F) and total oral clearance CL/F) was fitted for each. It was assumed that artesunate was completely converted to DHA for determination of the dose in the pharmacokinetic analysis of DHA and of the total anti-malarial activity. Due to the small number of detectable samples (at most 2 per subject, one after the first dose and one after the third dose) only one random effect could be fitted. Inter-subject variations in clearance and volume of distribution were examined separately and in the final model, the parameter with larger variation was fitted as a random effect. The effect of the dosing period after which sampling was done was examined by including a binary covariate (0 = sampling after dose 1/1 = sampling after dose 3) and was not accounted for by the random effects structure. Inter-subject variations in clearance and volume of distribution were examined separately and in the final model, the parameter with larger variation was fitted as a random effect.

In each model, the inter-subject variability in pharmacokinetic parameters was modelled with a log-normal error structure, for example: (CL/F_i_) = (CL/F) exp(η_i_^CL/F^), where CL/F_i _is the parameter value for the i^th ^individual, CL/F is the population mean, η_i_^CL/F ^is the random effect with zero mean and variance σ_CL/F_, which represents the inter-subject variability for the parameter. The magnitude of the inter-subject variability was expressed as a coefficient of variation (CV%) approximated by the square root of the variance estimate, while the residual variability was expressed as the standard deviation of the residual error. The variability in pharmacokinetic parameters was investigated by examining the effects of the following covariates: age, weight, temperature, respiratory rate, sex, logarithm of enrolment parasitaemia, presence of gametocytaemia on enrolment, drug formulation and day of measurement for DHA and total anti-malarial activity.

The log of the likelihood function was used to determine which covariates should be included in the model, in the forward selection procedure. The goodness of fit of each model was also assessed by the examination of the scatter plots of residuals versus predicted drug levels. The actual time of the sampling was used in the analysis. All compartmental analyses were performed using the S plus programme (SPLUS 6.0 for Windows, Mathsoft, Inc).

The final population models were used to calculate posterior estimates of AUC for each individual based on the actual dose received and based on the median total dose received in this population (AUC = dose/clearance). The AUCs were then compared between treatment arms by calculating their ratio and the 95% CI.

### Statistical methods

Data are summarized using medians and ranges. Continuous variables were compared between treatment groups using the Mann-Whitney test and categorical variables were compared using the chi-square test or Fisher's exact test, as appropriate.

Cure rates were estimated using the Kaplan-Meier method. Patients who developed new infections during the follow-up or were lost to the follow-up were censored in the analysis at the last visit. Patients with recurrent parasitaemia and no PCR results were excluded from the analysis. The difference in cure rates between treatments was calculated and the 95% confidence interval was estimated using Newcombe's method [16] and effective sample size [17].

## Results

Of the randomized patients, 64 (45.7%) were females and 76 (54.3%) males. The median (range) age of patients at baseline was 32 (7 – 59) months. There were no significant differences between treatment groups at baseline for all variables studied including demography (age, sex and ethnic group), clinical variables (weight, temperature and palpable spleen and liver), the measured parasitic variables and laboratory parameters. Demographic characteristics and clinical parameters at baseline of patients participating in the pharmacokinetic study are presented in Table 1.

**Table 1:** Baseline characteristics. Median (range) or number of patients (%).

	AS+AQ	AS/AQ
**Artesunate PK group**	N = 35	N = 35
Age (months)	37 (7 – 59)	32 (11 – 58)
Weight (kg)	14 (7 – 17)	12 (7.5 – 19)
Sex (n (%) males)	25 (71)	15 (43)
Temperature (°C)	39.0 (37.5 – 40.6)	38.7 (37.5 – 40.1)
Gametocytaemia (n (%) with gametocytes)	6 (17)	3 (9)
Hepatomegaly (n (%))	0 (0)	1 (3)
Splenomegaly (n (%))	1 (3)	1 (3)
Parasite count (/μL)	30,000 (2000 – 162,000)	29,000 (2000 – 393,000)
		
**Amodiaquine PK group**	N = 35	N = 35
Age (months)	26 (12 – 59)	29 (7 – 58)
Weight (kg)	12 (7 – 31)	12 (6 – 19)
Sex (n (%) males)	16 (46)	20 (57)
Temperature (°C)	38.4 (37.5 – 40.2)	38.4 (37.5 – 40.8)
Gametocytaemia (n (%) with gametocytes)	1 (3)	7 (20)
Hepatomegaly (n (%))	1 (3)	0 (0)
Splenomegaly (n (%))	1 (3)	2 (6)
Parasite count (/μL)	29,600 (1000 – 170,000)	27,000 (1000 – 468,000)

### Efficacy analyses

Five patients (all in the FDC group) had early treatment failure due to the development of severe malaria or danger signs between Day-0 and Day-3. Twenty-three patients had recurrent parasitaemia after Day-7: 12 from the AS/AQ group and 11 from the AS+AQ group. Seventeen of the 23 recurrent cases had PCR samples analysed, for the remaining patients the samples were missing. Of the 17 samples, eight (FDC AS/AQ) and four (AS+AQ) patients were classified by PCR as new infections and five were classified as recrudescent infections (two from the AS/AQ FDC group and three from the AS+AQ group).

Kaplan-Meier estimates of the cure rates (95% CI), after adjusting for PCR results and excluding patients with recurrent parasitaemia and no PCR result, were 0.946 (0.840–0.982) in the AS+AQ group and 0.892 (0.787 – 0.947) in the AS/AQ group. The estimated difference (95% CI) in the cure rates was 0.053 (-0.051 to 0.154).

### Clinical adverse events

By direct questioning, 87.1% (61/70) of patients in the FDC AS/AQ group and 85.7% (60/70) in the AS+AQ group reported at least one sign/symptom over the 28 days from a pre-defined list of 11 signs/symptoms (solicited adverse events: weakness, headache, vertigo, abdominal pain, anorexia, nausea, vomiting, diarrhoea, coughing, rhinitis, cutaneous rash). None of these were considered possibly or definitely related to the treatment. The most frequent (>20%) solicited adverse events were coughing, rhinitis, anorexia, diarrhoea and weakness.

There were three SAEs in the FDC AS/AQ treatment group: (i) a 36-month old boy who died of severe malaria on Day-0, (ii) a 21-month old girl who developed severe anaemia on Day-1 that resolved on Day-3 (iii) a 36-month old boy with gastroenteritis (salmonella) on Day-14 that resolved on Day-26. For the AS+AQ treatment group, a 26-month old girl had convulsions on Day-0 but subsequently recovered. All SAEs were considered unrelated to study drugs.

The boy who died was previously well and presented with a 24 hours history of fever and anorexia. On examination, he was alert, febrile (38.7°C, axilla) and had pale conjunctiva but was not jaundiced. His parasitaemia was 139,625/μL and his haemoglobin was 8.2 g/dl. An uncomplicated malaria was diagnosed and treated with paracetamol 250 mg and the FDC ASAQ 50 mg/135 mg. Five hours later, he went into a coma and he was transferred to hospital with a diagnosis of severe malaria. His temperature was 36.5°C; blood glucose 15.528 mg/dl, haemoglobin 6.9 g/dl and had a parasitaemia of 85,546/μL. He was treated with intravenous quinine but he died several hours later. The drug concentrations measured two hours after dosing were 620 ng/ml of DHA and 4.5 ng/ml of artesunate.

### Laboratory parameters

There were no differences between treatment groups with respect to values of the laboratory parameters on Day-0, Day-28 and with respect to their changes over time (all p > 0.05, Kruskal-Wallis test). From baseline to Day-28, the median (range) haematocrit increased from 26.5 (17.1 – 37.4)% to 32.0 (20.5 – 39.3)% and the median change was 3.9 (-3.3 – 15.6)%. The median (range) platelet counts rose from 173.0 (19.0 – 648.0) × 10^9^/L to 391.0 (56.0 – 906.0) × 10^9^/L, with the median (range) change of 218.0 (-242.0 – 692.0)/L.

Median Day-0 and Day-28 asparate aminotransferase (AST) values were 62.8 (27.3 – 448.6) and 54.4 (4.9 – 824.2) IU/L, respectively with median (range) change: -10.2 (-392.0 – 760.2) IU/L. Median total serum bilirubin fell from 15.7 (0.3 – 73.2) to 3.4 (0.0 – 97.0) μmol/L (median change: -10.0 [-68.6–85.2] μmol/L) and median serum creatinine fell from 42.3 (14.1 – 71.6) to 34.8 (18.1 – 105.7) μmol/L (median change: -7.3 [-34.7–34.0] μmol/L). Median serum alanine aminotransferase (ALT) values changed little over time (23.9 [6.6–174.8] to 25.2 [12.5–759.9] IU/L) with the median (range) of 0.8 (-149.6 – 724.5) IU/L.

One FDC group patient whose post Day-2 DeAQ concentration was 616.8 ng/ml developed CTC grade 3 (> 5 – ≤ 20 × ULN) liver enzymes during follow up (total bilirubin values normal): AST = 64 IU/L (D0), 294 (D14), 352 IU/L (D21) and 222 IU/L (D28). Corresponding ALT values 23, 389 and 227 IU/L. The patient's bilirubin levels were normal during the whole follow-up period. All other patients with DeAq levels > 600 ng/mL had normal biochemical results.

Severe neutropaenia (defined as a neutrophil count of <1000/μL) rates on Day-0 and Day-28 were 1/132 (0.8%) and 0/84 (0.0%), respectively. Leukopaenia (WBC <6000/μL for children <12 months, <5,000/μL for children between 1 and 5 years) rates on Day-0 and Day-28 were 2/135 (1.5%) and 0/85 (0.0%), respectively.

### Pharmacokinetic analysis

**Figure 1:**
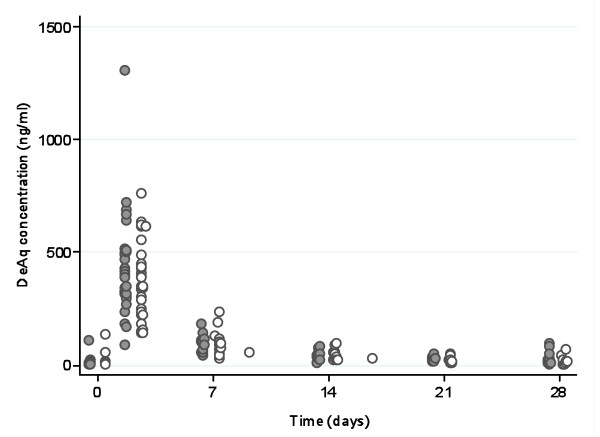
**Measured concentrations of Desethylamodiaquine**. Filled circles denote samples from patients in the FDC AS+AQ treatment arm and hollow circles denote samples from patients in the AS/AQ treatment arm.

**Figure 2:**
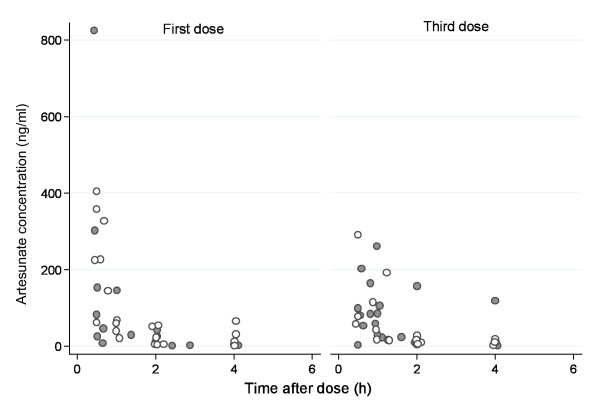
**Measured concentrations of Artesunate**. Filled circles denote samples from patients in the FDC AS+AQ treatment arm and hollow circles denote samples from patients in the AS/AQ treatment arm.

**Figure 3:**
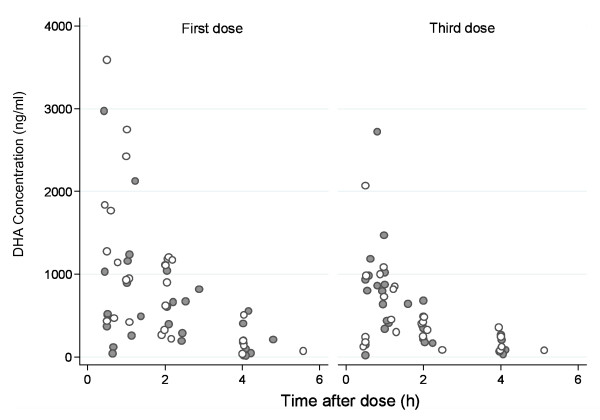
**Measured concentrations of DHA**. Filled circles denote samples from patients in the FDC AS+AQ treatment arm and hollow circles denote samples from patients in the AS/AQ treatment arm.

Measured concentrations of desethylamodiaquine, DHA, and AS in all patients are presented in Figures 1, 2 and 3; concentrations of AQ and DeAq are summarized in Table 2. Seven patients were excluded (four in the AS PK group and three in amodiaquine PK group) from the pharmacokinetic analysis since they vomited within one hour of study drug administration. Two further patients were excluded from the amodiaquine PK analysis since they had positive AQ and desethylamodiaquine samples before the first dose indicating a recent treatment with AQ and it was impossible to assess how much drug has already been absorbed.

**Table 2:** Plasma concentrations of amodiaquine and desethylamodiaquine

	Median (Range) (N) Concentration (ng/mL)	
	
	AS+AQ	AS/AQ
**Amodiaquine**		
Pre-dose 1	3.54 (n/a) (1)	1.96 (n/a) (1)
Post-dose 3	15.0 (3.8 – 48.9) (32)	14.1 (5.6 – 54.4) (30)

**Desethylamodiaquine**		
Pre-dose 1	7.7 (1.0 – 108.9) (15)	9.3 (1.6 – 137.1) (15)
Post-dose 3	396.6 (86.9 – 1307.8) (32)	347.1 (143.3 – 759.3) (30)
Day-7	73.9 (40.7 – 186.9) (15)	91.5 (31.4 – 235.2) (15)
Day-14	48.8 (11.7 – 84.1) (15)	41.0 (24.1 – 99.2) (16)
Day-21	29.0 (19.2 – 47.3) (10)	17.7 (9.3 – 48.0) (18)
Day-28	17.7 (5.4 – 100.1) (18)	17.4 (5.5 – 69.9) (12)

Positive desethylamodiaquine levels, but negative corresponding AQ levels, were detected in 28 patients (14 on each treatment arm) before the first dose, suggesting previous AQ administration. These levels were modelled assuming first order elimination of the previous dose, and subtraction from the study drug profile.

One child, who received a total dose of 39 mg/kg, had a very high plasma concentration of desethylamodiaquine recorded 4 hours after the last dose (1308 ng/mL). However, the plasma concentration on Day-7 (142.6 ng/mL) was within the range for other patients. This patient had a low desethylamodiaquine concentration before the first dose (18.5 ng/mL), which did not explain the subsequent very high level.

#### Amodiaquine

In total 60 samples (31 in the AS+AQ group and 29 in the AS/AQ group) had detectable levels of AQ, all taken at approximately 4 hours (median [range] = 4 [3.9–4.8]) after the third dose, at similar times in the two treatment groups (p = 0.35, Mann-Whitney test). These levels were not significantly different between treatment arms (p = 0.9, Mann-Whitney test) median (range): 15.5 (3.8 – 48.9) and 14.1 (5.6 – 54.4) ng/mL, respectively.

#### Desethylamodiaquine

**Figure 4:**
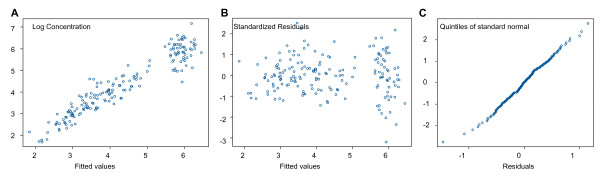
**Residuals plots for the desethylamodiaquine pharmacokinetic population model: (A) observed versus fitted values; (B) residuals versus the fitted values; (C) normal plot of residuals**.

The pharmacokinetics of desethylamodiaquine was best described by a two compartment open model with first order absorption and elimination. The best fit was obtained with concentration modelled on a log scale and homoscedastic error model. The final estimates of the pharmacokinetic parameters are presented in Table 3. Figure 4 shows residuals plots. A good agreement between fitted and observed concentrations was achieved, although there were a few outliers with very high concentrations (Figure 4A). There were no obvious patterns in the residuals (Figure 4B), which satisfied the normality assumption (Figure 4C). Inclusion of any of the covariates, including drug formulation, into the population pharmacokinetic model did not reveal any statistically significant associations with clearance.

**Table 3:** Estimated pharmacokinetic parameters for desethylamodiaquine

Parameter	Estimate (SE)	Inter-subject variation CV%
Ka (h^-1^)	0.13 (fixed)	
CL/F (L kg^-1 ^h^-1^)	0.610 (0.038)	22.7%
Q (L kg^-1 ^h^-1^)	0.680 (0.306)	
V_central_/F (L kg^-1^)	35.4 (11.5)	
V_peripheral_/F (L kg^-1^)	87.9 (17.1)	
T 1/2 (days)	9.0 [7.3–11.6]^1^	
σ	0.457	

^1^range of predicted values;

Ka – absorption rate constant; CL/F – total clearance;

Q – inter-compartmental clearance; V_central_/F – volume of the central compartment;

V_peripheral_/F – volume of the peripheral compartment; F – fraction of drug absorbed;

σ – residual additive error on the log_e _scale.

There were no differences in the distribution of the posterior individual estimates of total oral clearance (CL/F) between the two treatment arms: mean (standard deviation, CV%) 0.61 (0.11, 18%) L/kg/h in the loose formulation arm and 0.63 (0.10, 15%) L/kg/h in the fixed formulation arm; difference 0.024 (95%CI: -0.077 to 0.030) L/kg/h; normal distribution for the estimates could be assumed. This reflected very similar AUCs in the two treatment groups, normalized for an oral dose of 34 mg/kg; median (range) of 57.76 (40.00 – 82.32) ng/mL.h with the loose formulation arm and 54.75 (40.73 – 73.64) ng/mL.h with the fixed formulation. The ratio of the loose formulation AUC to the fixed formulation AUC was estimated as 1.043 (95% CI: 0.956 to 1.138).

However, the actual dose received was approximately 10% higher in the loose combination arm so the observed AUCs were also higher: median (range) of 62.5 (24.4 – 95.3) ng/mL.h in the loose formulation group and 48.7 (39.0 – 92.3) ng/mL.h in the fixed formulation group (p < 0.001).

**Figure 5:**
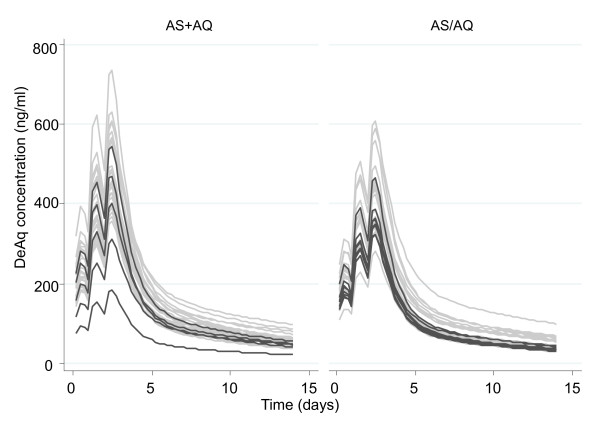
**Predicted desethylamodiaquine (DeAq) concentrations from the population model**. Dark grey curves represent profiles for patients with recurrent parasitaemia.

**Figure 6:**
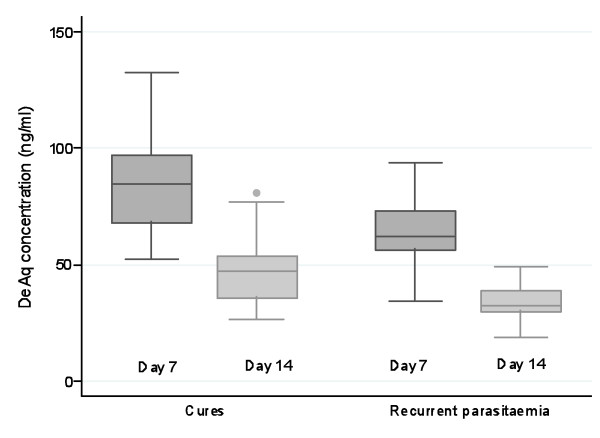
**Predicted desethylamodiaquine (DeAq) levels at day-7 and day-14 for patients who were cured and those who had recurrent parasitaemia**.

In total fourteen patients in the group of patients with measured desethylamodiaquine levels had recurrent parasitaemia: nine in the fixed dose combination group (two recrudescences, six new infections and two with no PCR results) and five in the loose combination group (three recrudescences, one new infection and one with missing PCR). Based on pharmacokinetic models these patients had relatively low predicted concentrations of desethylamodiaquine (Figure 5). Predicted concentrations at Day-7 and Day-14 were significantly lower (p < 0.001 for both, Kruskal-Wallis test) in patients with recurrent parasitaemia (Figure 6). Among patients with a recurrent parasitaemia, seven patients had measured drug concentrations on Day-7 and another seven patients on Day-14. Five of the measurements (71%) on Day-7 (median [range]: 53.2 [41.2–74.1] ng/mL) were below the median value for the non-recurrent cases (median [range]: 95.1 [31.4–235.2] ng/mL, n = 23). The other two cases with concentrations (101.3 and 190.8 ng/mL) above the median were reinfections. Day-7 DeAq concentration was measured in three out of five patients with PCR confirmed recrudescence and they were all below 75 ng/mL giving an estimate of the cure rate in patients with Day-7 concentrations above 75 ng/mL as 100%. For the estimated Day-7 concentrations, only one (out of 5) patient with PCR confirmed recrudescence has the value above 75 ng/mL (93 ng/mL), which gives the corresponding cure rate of 97%.

**Figure 7:**
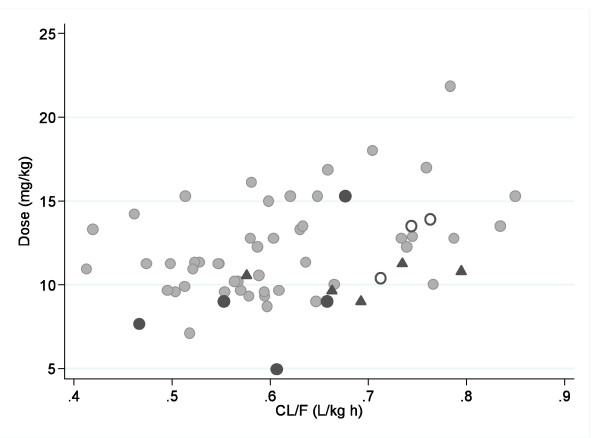
**Estimated desethylamodiaquine clearance and amodiaquine dose received for: grey circles – patients without recurrence of the parasitaemia; dark circles – patients with recrudescence; triangles – patients with reinfections and empty circles – patients with recurrent infection without a PCR result**.

Four of the seven (57%) measurements on Day-14 (median [range]: 25.4 [11.7–84.2] ng/mL) were below the median value for the non-recurrent cases (median [range] 48.8 [25.2–99.2] ng/mL, n = 23), including the only PCR confirmed recrudescence in this group. Four out of five patients with PCR confirmed recrudescence received doses below 10 mg/kg (4.9, 7.7 and two patients 9.0 mg/kg) and their estimated clearance was at the 54^th^, 7^th^, 28^th ^and 67^th ^percentile, respectively (Figure 7). The fifth patient, with a high dose (15.3 mg/kg), had relatively rapid clearance at the 73^rd ^percentile.

#### Artesunate and DHA

**Figure 8:**
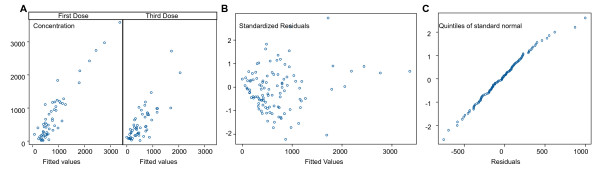
**Residuals plots for DHA pharmacokinetic population model: (A) observed versus fitted values; (B) residuals versus the fitted values; (C) normal plot of residuals**.

**Figure 9:**
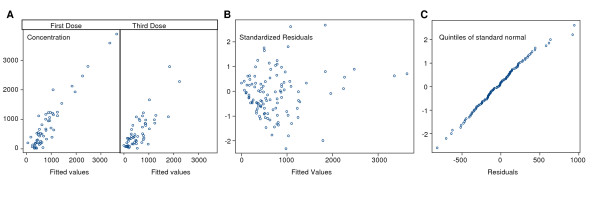
**Residuals plots for pharmacokinetic population model for the total anti-malarial activity: (A) observed versus fitted values; (B) residuals versus the fitted values; (C) normal plot of residuals**.

The pharmacokinetics of DHA and the total anti-malarial activity were best described by a one compartment model with volume of distribution fitted as a random effect, on the original scale and homoscedastic error model (Table 4). There was very little variation between subjects in estimated total oral clearance (standard deviation <10^-7^), but modelling clearance as a fixed parameter which differed with dosing periods improved the model significantly (p < 0.001 for both). This was justified by previous studies which have shown disease related changes in the pharmacokinetics of the AS derivatives [18,19]. Some of the between subject variation in volume of distribution could be explained by age or weight (p < 0.001 for each, likelihood ratio test) but the effect of these two covariates was not independent (Table 4). Inclusion of any other covariates, including drug formulation and dosing period, into the population pharmacokinetic model did not reveal any statistically significant associations with volume of distribution. Plots of fitted and observed concentrations show a reasonable agreement (Figures 8A and 9A). There were no obvious patterns in residuals (Figures 8B and 9B) and they satisfied the normality assumption (Figures 8C and 9C).

**Table 4:** Estimated dihydroartemisinin and total anti-malarial activity pharmacokinetic parameters.

Parameter	Estimate (SE)	Inter-subject variation CV%
**DHA**		
Ka (h ^-1^)	4.271 (2.091)	
CL/F^1 ^(L kg^-1 ^h^-1^)	0.636 (0.075)	
Increase in CL/F due to the 3^rd ^dose period	0.760 (0.160)	
V/F (L kg^-1^)	2.285 (0.317)	47%
Effect of Age on V/F	0.063 (0.015)	
T 1/2 (h)		
1^st ^dose	2.5 [0.8–4.5]^2^	
3^rd ^dose	1.1 [0.4–2.0]^2^	
σ (ng/mL)	340	
		
**Anti-malarial Activity**		
Ka (h^-1^)	4.744 (2.587)	
CL/F^1 ^(L kg^-1 ^h^-1^)	0.616 (0.071)	
Increase in CL/F due to the 3^rd ^dose period	0.662 (0.150)	
V/F (L kg^-1^)	2.134 (0.287)	48%
Effect of Age on V/F	0.063 (0.013)	
T 1/2 (h)		
1^st ^dose	2.4 [0.5–5.7]^2^	
3^rd ^dose	1.2 [0.2–2.7]^2^	
σ (ng/mL)	353	

^1^population mean estimate of CL/F for the first dose period

^2^90% range of predicted values;

Ka – absorption rate constant; CL/F – total clearance; V/F – volume of distribution; F – fraction of drug absorbed; σ – residual additive error in DHA units

Since no differences in clearance for both DHA and anti-malarial activity were found between treatment arms (p = 0.73 and p = 0.71 respectively), the corresponding AUCs were estimated as 4.29 (95% CI = 3.48 – 5.59) ng/mL.h and 4.43 (95% CI = 3.61 – 5.75) ng/mL.h after the first dose for all patients, assuming a 3.7 mg/kg dose of AS. After the third dose the clearance approximately doubled so the AUCs were approximately halved: 1.96 (95% CI = 1.47 – 2.93) ng/mL.h and 2.14 (95% CI = 1.59 – 3.25) ng/mL.h, for the same dose.

## Discussion

For diseases requiring treatment with a combination of drugs, fixed combinations (FDC) are increasingly considered the optimum therapeutic approach. It is essential, therefore, to confirm that the bioavailability of any newly developed FDC is similar to that of the previously established and validated loose drug combination in the disease being treated.

This population pharmacokinetic evaluation of two AS-AQ regimens – one comprising loose tablets, and the other a newly developed fixed dose co-formulation – indicates that the two formulations have similar pharmacokinetic properties in young children with acute malaria, the main target group for this treatment. Amodiaquine was converted rapidly to desethylamodiaquine presumably by hepatic metabolism in a "first pass" and so it is the metabolite, which contributes most of the anti-malarial activity [20,21]. The estimated desethylamodiaquine half life of 9 (7 – 12) days is in broad agreement with previously published estimates from studies in children with falciparum malaria: median (range) of 7.4 (3.3 – 30.5) days [22] from the individual patients analysis; range 3 – 12 days from the population analysis [14]. Population analysis of sparse data, in general, produces narrower ranges of estimates since the extreme estimates observed in the individual patient analysis are shifted towards the population mean. Modelling based on sparse data may also underestimate very long terminal elimination phases at low concentrations (although these may not be of relevance to the immediate anti-malarial effect). There were no significant differences in the main pharmacokinetic parameter estimates between formulations, the ratio of AUC loose to fixed formulation was 1.043 (0.956 – 1.138) for a normalized total oral dose of 34 mg/kg.

In this study, measured concentrations of desethylamodiaquine had more symmetrical distributions and narrower ranges in the fixed dose combination but this does not reach statistical significance. This is in line with the observations made in the comparative pharmacokinetic study in healthy volunteers in which it appears that the FDC generates less variable results (P. Olliaro, V. Navaratnam; personal communication).

Artesunate is converted rapidly to dihydroartemisinin (DHA) *in vivo *and most of the anti-malarial activity derives from the metabolite. The pharmacokinetic parameter estimates of DHA and the total anti-malarial activity, defined as the sum of the molar equivalent plasma levels of DHA and AS, indicated rapid absorption and elimination. As noted in previous pharmacokinetic studies [23] plasma levels were significantly higher following the first dose when the patient was acutely ill than after subsequent doses when the patient was usually afebrile and clinically improved. AUC and elimination half life were halved for the third dose compared to the first dose. The bioavailability from the co-formulated product was similar to that of the separate tablets. The higher rate of early treatment failures observed in the FDC group is not confirmed neither in the efficacy study of children in Burkina Faso (Sirima, 2009) nor in another multicenter study of the FDC (Ndiaye, 2009). Both studies also showed similar parasite clearance rates on Day-2 in the two treatments groups. Poor drug absorption cannot be confirmed in the five children with early treatment failure. Drug concentrations were available in two children: DHA level of 1202 ng/ml in one child measured at 2.1 hours and DHA level of 620 ng/ml and artesunate level of 4.5 ng/ml in the second child measured at 2 h. These drug levels are comparable with values for other children (Figures 2 and 3). However, the sparse nature of the data, the short half lives of the drugs and the limitations of the population modeling unable us to examine individual profiles and their relation with the treatment outcome properly.

Associations were identified between outcome and desethylamodiaquine levels on Day-7 and 14. Predicted levels at Day-7 and Day-14 were significantly lower (p < 0.001) in patients with recurrent parasitaemia. For measured concentrations, the association was less clear, probably because of the low number of patients with plasma levels available, but all patients with PCR confirmed recrudescent parasitaemia or with recurrent parasitaemia and missing PCR results had Day-7 concentrations below 75 ng/mL giving 100% cure rate in patients with Day-7 concentrations above 75 ng/mL. This cure rate was estimated as 97% based on the estimated Day-7 concentrations.

There was no indication of a relationship between high blood concentrations of either drug and adverse effects. Of the 10 patients with DeAq blood levels higher than 600 ng/ml post 3^rd ^dose, only one had evidence of hepatocellular injury that was probably AQ related and was resolving by Day-28. Transient, AQ induced hepatocellular liver toxicity is well described and is probably a type II immune reaction.

Although rates of absorption were not examined in detail because of the sparse sampling, there were no obvious major differences in the observed concentrations collected during the absorption phase for the two drug formulations. The elimination kinetics estimated from sparse data may have missed a slower elimination phase and, therefore, underestimated the terminal elimination phase, but this is unlikely to contribute significantly to the therapeutic response. Importantly, the estimated bioavailability from the co-formulated product was similar to that of the separate tablets. Thus, if there is a longer terminal phase of elimination, it is unlikely to start at different concentrations with the two formulations.

## Conclusion

These data support the pharmacological equivalence of the new fixed dose AS/AQ formulation with the previously established separate products. The likely improved adherence associated with this relatively well tolerated, simply administered, stable (shelf life in tropical conditions 3 years), inexpensive (cost below 0.5/1 USD for child/adults for 3-day treatment) FDC should be a significant operational advantage.

## List of abbreviations used

ACT: Artemisinin Combination Therapy; AQ: Amodiaquine; AS: Artesunate; AS/AQ: Artesunate and Amodiaquine in a fixed dose combination; AS+AQ: Artesunate and Amodiaquine in a loose dose combination; DHA: Dihydroartemisinin; DeAQ: Desethylamodiaquine; FDC: Fixed-dose combination.

## Conflict of interests

NJW is co-chairman of the WHO Global Malaria Programme technical expert group on the prevention and treatment of malaria. This clinical study was funded by the Research Directorate General of the European Commission (Contract N° ICA4- CT-2002610046) and the Drugs for Neglected Diseases initiative.

## Authors' contributions

KS analysed the data and drafted the manuscript, NJW contributed to the design of the study, data analysis, and manuscript writing. JRK was the project manager for the development of the ASAQ FDC and for the specific study, report and publication. WT was the clinical manager of the study, was part of the protocol development team and reviewed the data and the first draft of the manuscript. SS was the Principal Investigator, was also part of the protocol development team, managed the clinical team in Burkina Faso and reviewed the first draft of the manuscript. JAS designed the PK schedule and contributed to the manuscript writing. CCM analysed the clinical data and was responsible for the clinical study report.
